# Radiological Outcomes, Complications, and the Influence of Risk Factors in PHILOS Repair of Three- and Four-Part Proximal Humerus Fractures with and Without Femoral Head Allograft: Insights from a Cohort of 116 Patients

**DOI:** 10.3390/jcm15020910

**Published:** 2026-01-22

**Authors:** Zoltan Cibula, Milan Cipkala, Diaa Sammoudi, Marian Grendar, Monika Cervencova

**Affiliations:** 1 Jessenius Faculty of Medicine in Martin, Comenius University Bratislava, 03659 Martin, Slovakia; cibulazolo@gmail.com (Z.C.); marian.grendar@uniba.sk (M.G.); 2 Department of Orthopaedic Surgery, University Hospital Martin, Kollarova 2, 03659 Martin, Slovakia; milan.cipkala@gmail.com (M.C.); dia.samoudi@gmail.com (D.S.)

**Keywords:** proximal humerus fracture, PHILOS, allograft, complications

## Abstract

**Background**: Complications after proximal humerus osteosynthesis are not uncommon. The aim of this study was to compare the outcomes of osteosynthesis using PHILOS with fresh-frozen femoral head allograft augmentation and without it, and to assess the influence of risk factors and their impact on the occurrence of postoperative complications. **Methods**: This retrospective study evaluates the radiological outcomes and complications of treating proximal humerus fractures (Neer III–IV) in 116 patients over 50 years of age treated between 2017 and 2021. **Results**: Osteosynthesis without allograft was performed in 84 patients and with allograft in 32 patients. In total, 42 patients (36%) had a three-part fracture and 74 (64%) had a four-part fracture. The Deltoid Tuberosity Index was comparable between the groups (1.59 ± 0.25 vs. 1.50 ± 0.26; *p* = 0.802). The average duration of surgery was 101.3 ± 21.3 min with allograft and 86.0 ± 31.9 min without allograft (*p* = 0.004). AVN was verified in four patients (3.5%), head collapse in nine (8%), cut-out in six (5%), reoperation in eight (7%), infection in three (2.5%), and pseudoarthrosis in one (1%) case. **Conclusions**: An allograft augmentation improves construct stability, but cannot compensate for inadequate surgical technique. None of the risk factors significantly influenced the development of AVN and pseudoarthrosis. The greater tubercle comminution (*p* = 0.005), calcar loss (*p* = 0.020, *p* = 0.112), allograft augmentation (*p* < 0.001), and medial hinge restoration (*p* = 0.012, *p* = 0.002) were significant risk factors associated with HC and screw cut-out, respectively. The greater tubercle redislocation was influenced by its comminution, calcar loss, and the use of allograft augmentation. HFZ and DTI had no significant impact on surgery results or complications.

## 1. Introduction

Proximal humerus fractures are common fractures in older patients which are related to age-related deterioration of bone quality due to osteoporosis. The method of treatment depends on the type and displacement of the fracture, as well as the patient’s age and functional demands. Most stable, minimally displaced fractures heal conservatively with a good functional outcome; however, approximately 20% of cases are unstable and require surgical treatment. Traditional methods of Open Reduction and Internal Fixation (ORIF) using angular-stable plates or intramedullary implants are associated with complications such as loss of reduction, screw penetration, or avascular necrosis. Reverse Shoulder Arthroplasty (RSA) achieves better functional outcomes and a lower reoperation rate in older patients with complex three- and four-part fractures [1], but even this method does not guarantee full functional restoration and quality of life. Bone quality and defect in the epi-metaphysis of the proximal humerus remain significant limiting factors for the success of osteosynthesis. The use of a bone graft is a suitable method for open reduction and plate osteosynthesis, which improves the possibilities of reduction and overall stability. The main benefit of a bone graft is mechanical support for the medial column, reducing the risk of varus collapse and secondary displacement. The most commonly used are fibular allografts [2,3,4] or femoral head allografts [5,6]. Autologous bone can also be an option. Indication for allograft in the treatment of acute proximal humerus fractures in the elderly remains controversial [7]. The aim of this retrospective study is to compare the radiological outcomes of proximal humerus osteosynthesis using an angular-stable PHILOS plate (DePuy Synthes) without the use of a femoral head bone allograft or with its use. In addition, this study aims to assess the influence of risk factors and their impact on the occurrence of postoperative complications.

## 2. Materials and Methods

This study retrospectively evaluated 181 patients with proximal humerus fracture treated with Philos plate between January 2017 and March 2021. Based on the exclusion criteria, a final group of 116 patients with three- and four-part proximal humerus fractures according to the Neer classification was created. Patients were treated with PHILOS (DePuy Synthes, Johnson & Johnson MedTech, Zuchwil, Switzerland) plate osteosynthesis.

The cohort was divided into two groups. The first group consisted of patients in whom spongioplasty of the bone defect was not used. The second group included patients in whom the bone defect in the meta-epiphysis of the proximal humerus was filled with a bone allograft. In all cases, the bone graft used was a fresh frozen femoral head allograft.

**Figure A1:**
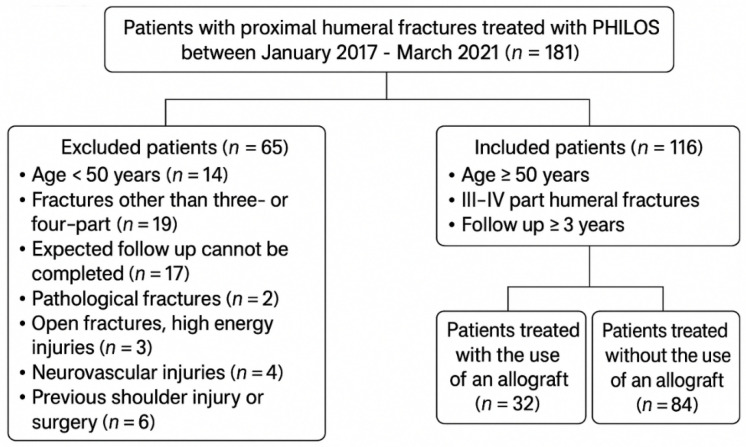
Flowchart of patient recruitment and follow up.

Inclusion criteria for the study cohort were patient age over 50 years, a three- or four-part proximal humerus fracture, and a minimum follow-up period of three years. The mean follow-up was 42 ± 3 months. An exception to the follow-up period was made for patients who experienced severe complications during treatment, including head collapse, avascular necrosis, or osteosynthesis failure, and required reoperation. Exclusion criteria included high-energy injuries, fractures with neurovascular injury, open fractures, pathological fractures, and previous injuries or surgeries of the shoulder joint. Based on the Neer classification, we excluded patients with fractures other than three- and four-part fractures. Children and young patients with good bone quality were not included in the cohort. Patients whose evaluated parameters could not be adequately assessed by radiological examination were also not evaluated. A flowchart of patient enrollment and follow-up, illustrating the main steps of the study design from participant recruitment and the application of inclusion/exclusion criteria to the final selection of the sample, is presented in the Appendix A, Figure A1.

Osteosynthesis of the fractures was performed in the acute phase, being, on average, 3.8 days after the injury. Patients were routinely positioned in the beach chair position, which allows for free movement of the shoulder, optimal approach, and visualization of the proximal humerus in both AP and axial projections. The surgical approach was chosen according to the surgeon’s preference: either the deltopectoral approach or the anterolateral approach according to Mackenzie.

Neutralization sutures are applied to the main parts of the rotator cuff to aid reduction. These are fixed to the PHILOS plate holes as part of the complete osteosynthesis. Based on the results of imaging examinations and intraoperative findings, the decision to use a bone graft (fresh frozen femoral head allograft) was made individually, depending on the extent of the defect in the epi-metaphyseal region, fracture type, and bone quality. Optimally, we applied the PHILOS plate to the lateral side of the humerus 2–4 mm lateral to the bicipital groove and 5–7 mm below the top of the greater tuberosity.

The femoral head was divided into smaller parts, and a graft of optimal shape and size was modeled from the central part during the surgical procedure to precisely fill the defect in the epi-metaphysis of the proximal humerus. The graft prepared in this way serves as an aid in reducing the humeral head and simultaneously provides additional medial structural support to the PHILOS plate osteosynthesis. The bone allograft is implanted through the fracture line dorsal to the bicipital groove. After thorough reduction in the tuberosities and the humeral head, the osteosynthesis was completed.

All patients received a prophylactic dose of antibiotics for 24 h, and in the case of allograft implantation, local antibiotic prevention was also used. Commonly used antibiotics include cefazolin (Vulmizolin^®^, 2 g; BB Pharma s.r.o., Martin, Slovakia) or, in the case of confirmed β-lactam allergy, clindamycin (Klimycin^®^, 600 mg i.v.; Medochemie Ltd., Limassol, Cyprus) was used. Intraoperatively, the bone allograft was first rehydrated and thawed in sterile physiological saline solution (NaCl 0.9%) with the addition of gentamicin (Gentamicin B. Braun 80 mg/2 mL, B. Braun Melsungen AG, Melsungen, Germany) at a concentration of 40–80 mg/L. Subsequently, the bone graft was rinsed three times in the antibiotic solution after preparation and before the actual implantation.

Under the supervision of a physiotherapist, patients began pendulum exercises of the shoulder and active exercise of peripheral joints on the first postoperative day. After three weeks, assisted passive and active range of motion exercises for the shoulder were introduced, with a transition to active exercises in the full range of shoulder motion after six weeks.

The basic evaluated parameters of the patient cohort were their age, sex, and the aforementioned use of bone allograft, timing of the surgical procedure, and duration of the operation between the patient groups. Complications assessed up to three years after surgery included avascular necrosis, humeral head collapse, fragment dislocation, cut-out, pseudoarthrosis, infection, and the need for reoperation. The influence of individual risk factors on the incidence of complications was also evaluated. Therefore, targeted statistical analyses were performed to determine which preoperative or intraoperative variables significantly influence the final outcome, as reflected by the presence or absence of complications. The evaluated risk factors included the following: patient age and sex, fracture pattern, timing of the surgery, interruption of the medial hinge, comminution of the greater tubercle, valgus/varus deformity, the use of a bone graft, and medial hinge restoration. We also evaluated age, DTI (Deltoid Tuberosity Index), and HFZ (Head Fragment Size) as independent risk factors.

**Figure 1:**
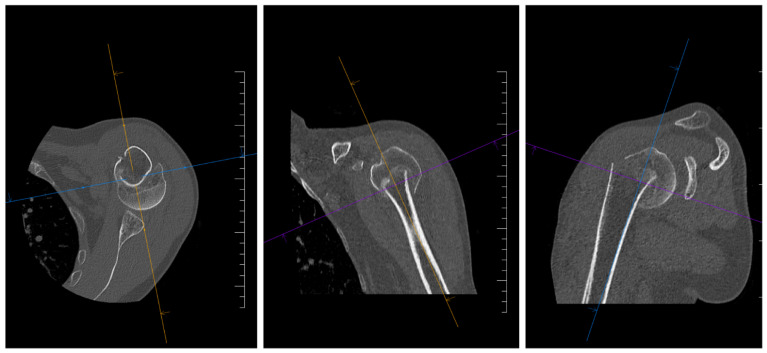
CT imaging of a three-part proximal humerus fracture during preoperative planning.

The fracture was diagnosed based on a standard X-ray examination in anteroposterior (AP) and lateral projection, which was supplemented by a CT examination of the affected shoulder (Figure 1).

**Figure 2:**
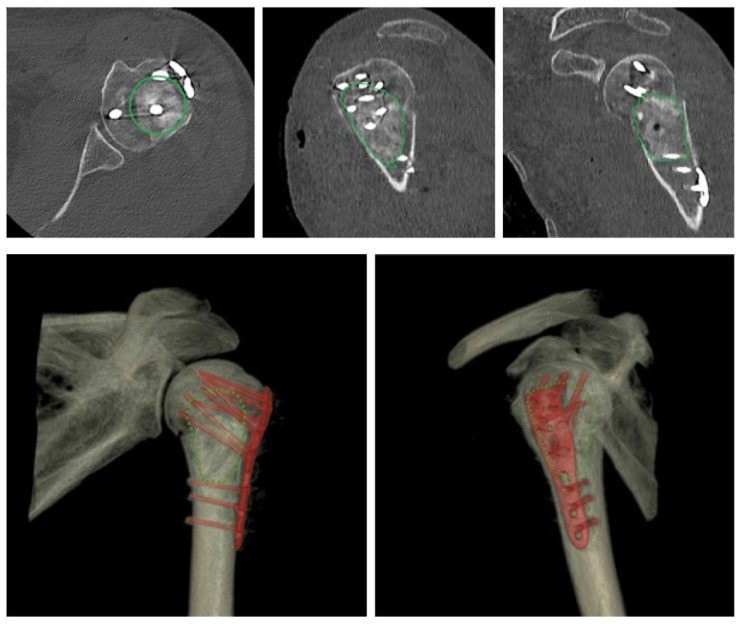
CT imaging of a three-part proximal humerus fracture after surgical treatment using a PHILOS plate with the use of a bone allograft. Imaging in standard planes and 3D reconstruction showing the bone allograft.

Based on the results of the imaging, the fracture was classified according to the Neer classification of proximal humerus fractures. Before the surgical procedure itself, the type of dislocation (valgus/varus), dislocation of the main fragments, calcar humeri, and deltoid tuberosity index (DTI) were evaluated. The size of the head fragment was determined based on the CT examination, with the measurement performed using the HFZ (Head Fragment Size) method. Postoperative images (at an interval of 1–6 months after surgery) were evaluated in terms of the quality of reduction in the greater tuberosity fragments, calcar humeri, humeral head, and the position of the PHILOS plate (Figure 2). Suboptimal position or loss of reduction after the surgical procedure was evaluated as any dislocation, rotation, or change in the position of the main fragments of >4 mm and an angular deformity of more than 10°. Suboptimal plate position was evaluated as proximal or distal placement of the PHILOS plate by more than 3 mm, causing impingement, or a position that does not allow for adequate screw fixation into the humeral head.

We evaluated bone quality by radiological measurement of the deltoid tuberosity index (DTI), defined as the ratio α/β. The value α represented the distance between the outer cortical edges just above the tuberositas deltoidea, and the value β represented the distance between the inner cortical edges at the same level. A DTI value of <1.4 was interpreted as cortical bone thinning due to osteoporosis or poorer bone quality. Conversely, a DTI value of >1.4–1.5 was interpreted as preserved cortical bone thickness and thus better bone quality.

**Figure 3:**
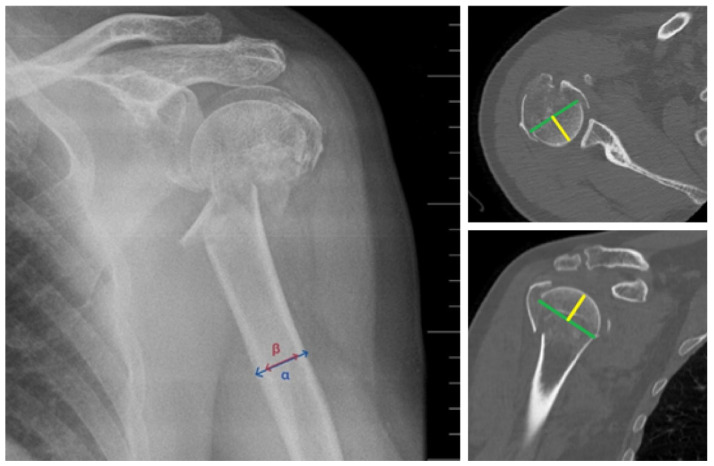
Measurement of the deltoid tuberosity index-DTI (**left**), as the ratio α/β. Evaluation of Head Fragment Size by measuring the remaining thickness of the humeral head in the axial (**top right)** and sagittal plane (**bottom right**).

Head Fragment Size (HFZ) is an evaluation parameter for quantifying the width of the remaining head fragment. The measurement is performed via CT examination in the axial and sagittal planes, where two values representing the width of the head fragment in the respective planes are recorded. The average value of these two measurements represents the resulting average HFZ, which objectively expresses the amount of bone mass remaining in the head and available for screw fixation (Figure 3).

The radiological measurements (Neer classification, dislocation—valgus/varus, dislocation and redislocation of the main fragments and calcar humeri, DTI, HFZ, AVN, cut-out, head collapse, non-union) were performed by an experienced radiologist specializing in orthopedics and trauma surgery who was not included in the study itself. All measurements were performed by this single individual. A subset of 20 randomly selected X-rays was re-evaluated after two weeks (M.C. and M.G.) to assess intra- and inter-observer reliability using intraclass correlation coefficients (ICC). ICC values of >0.80 were considered excellent.

The study does not evaluate functional outcomes (mobility, pain).

Data were reviewed and analyzed using Jamovi software (version 2.6.44.0), which is based on R [7,8,9]. Depending on the data distribution, the *t*-test or Mann–Whitney U-test was used for the analysis of continuous variables. Association between two categorical variables was tested using the chi-square test and Fisher’s exact test. Statistical differences were considered significant if the *p*-value was ˂0.05.

## 3. Results

The cohort retrospectively included 116 patients with a proximal humerus fracture, 75 women (65%) and 41 men (35%), with a mean age of 67 years (54–89 years). The average age of patients treated without the use of an allograft was 69 ± 10.1 years, while in the group where an allograft was used, it reached 65 ± 10.8 years. The difference in mean age was 4 years, and no statistically significant difference was found between the two groups (*p* = 0.714). Osteosynthesis using a PHILOS plate without allograft was performed in 84 patients (72%), while in 32 patients (28%), the bone defect was filled with an allograft.

According to the Neer classification, 42 patients (36%) with three-part and 74 patients (64%) with four-part fractures were treated in the cohort. In the group of patients with three-part proximal humerus fractures, 39 patients (46%) were treated without the use of an allograft, while 3 patients (9%) were treated with the use of an allograft. In the group with four-part fractures, 45 patients (54%) were treated without allograft and 29 patients (91%) were treated with the use of an allograft. These data indicate a more frequent use of allograft in more complex, four-part fractures. Valgus dislocation of the fracture after the injury was recorded in 65 patients (56%), while varus dislocation occurred in 33 patients (28%). Optimal PHILOS plate position was achieved in 112 patients (97%), while in 4 patients (3%), the position was evaluated as suboptimal (*p* = 0.906). The reduction in the greater tuberosity was non-anatomical in thirteen cases: four cases (13%) in patients treated with allograft, and nine cases (11%) in patients without allograft. The difference between the groups was not statistically significant (*p* = 0.785). The calcar humeri was anatomically reduced in 102 patients, and non-anatomical reduction was present in fourteen cases, with eight cases (25%) being patients with allograft and six cases (7%) being patients without allograft; the difference was statistically significant (*p* = 0.008). The average operative time in the cohort was 90 min. The duration of the operation was on average 101.3 ± 21.3 min in the group with allograft, while in the group without allograft it was 86.0 ± 31.9 min, which represented a statistically significant difference (*p* = 0.004). In the group of patients in whom no allograft was used, the operation was performed on average 3.5 ± 2.9 days after the injury, while patients with allograft were operated on 4.7 ± 2.4 days after the injury; the difference was statistically significant (*p* = 0.007). The Deltoid Tuberosity Index was comparable between both groups (without vs. with allograft), and no statistically significant difference was found (1.59 ± 0.25 vs. 1.50 ± 0.26; *p* = 0.802) (Table 1).

Complications occurred in both groups. Avascular necrosis was verified in four patients (3.5%), occurring in two cases in each group, without a statistically significant difference (*p* = 0.307). Humeral head collapse was observed in nine patients overall: in seven cases (22%) treated with allograft and in two patients (2%) without allograft; the difference was statistically significant (*p* < 0.001). Cut-out occurred in six patients (19%) in the group with allograft, while it did not occur in the group without allograft, which was statistically significant (*p* < 0.001). Screw loosening was recorded in five patients, three (9%) with allograft and two (2%) without allograft, with the difference not being statistically significant (*p* = 0.097). Reoperation was necessary in eight patients (7%), with an even distribution in both groups (*p* = 0.142)

In the cohort of 116 patients with proximal humerus fracture, preoperative and intraoperative variables were determined and their influence on the incidence of postoperative complications was monitored.

We assessed the following variables as risk factors for the development of specific complications: patient age, sex, fracture pattern according to Neer classification, timing of surgery, disruption of the medial hinge, greater tuberosity comminution, valgus/varus displacement, the use of a bone allograft, and restoration of the medial hinge.

None of the observed risk factors significantly contributed to the development of AVN in the studied cohort. Humeral head collapse demonstrated statistically significant associations with interruption of the medial hinge (*p* = 0.020), comminution of the greater tubercle (*p* = 0.005), and with both the use of a bone allograft (*p* < 0.001) and medial hinge restoration (*p* = 0.012). In this cohort, patient sex, age, and fracture pattern had a non-significant effect on the occurrence of humeral head collapse.

Significant risk factors for cut-out were comminution of the greater tubercle (*p* = 0.005) and the use of a bone allograft (*p* ˂ 0.001), as well as medial hinge restoration (*p* = 0.002). Non-significant effects on the occurrence of cut-out came from sex, fracture pattern, interruption of the medial hinge, and type of deformity.

In the patient cohort, redislocation of the greater tubercle was associated with comminution of greater tubercle (*p* = 0.010) and interruption of the medial hinge (*p* = 0.027), as well as the use of a bone allograft (*p* = 0.007).

**Figure 4:**
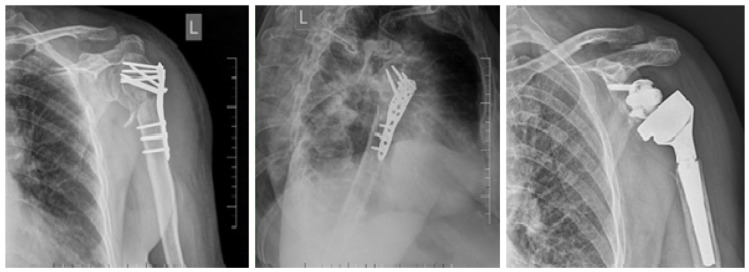
Failure of osteosynthesis with cut-out and varus collapse. Extraction of the PHILOS plate and implantation of a reverse shoulder arthroplasty.

In six patients, a reverse shoulder arthroplasty was implanted due to treatment complications, regardless of the use of a bone allograft (Figure 4).

Infection was confirmed in two patients with the use of allograft and in one patient without the use of allograft. Of the observed variables, medial hinge restoration demonstrated statistical significance in relation to the incidence of infectious complications.

Pseudoarthrosis of the surgical neck was confirmed in one patient, in whom an allograft was not used, and no significant influence of any of the observed risk factors on pseudoarthrosis development was confirmed. More information about the impact of individual risk factors and the incidence of postoperative complications can be found in Table 2.

Statistical analysis of the patient cohort treated with PHILOS, with and without the use of a bone allograft, unexpectedly indicated a higher incidence of humeral head collapse and screw cut-out in the allograft group, as well as a higher incidence of redislocation of the greater tuberosity. Therefore, we performed a more detailed evaluation to determine the underlying reasons, which was carried out on a selected group of patients with complications. We identified associations with medial hinge interruption and four-part fractures. Additionally, we report associations with inadequate reduction in the humeral calcar, varus malalignment, and calcar screw positioning.

Humeral head collapse in the subgroup of seven patients treated with a bone allograft occurred exclusively in cases with medial hinge interruption, and in six of these cases, the patients sustained four-part fractures. A more detailed analysis showed that none of the patients had anatomically reduced humeral calcar, absence of varus displacement, and simultaneously adequate positioning of the calcar screws.

Cut-out in the subgroup of six patients treated with a bone allograft occurred in five cases with medial hinge interruption, and in four of these cases, the patients sustained four-part fractures. In only one case within this subgroup was the humeral calcar anatomically reduced and the calcar screws correctly positioned, with no varus malalignment of the humeral head present; however, this case involved a three-part fracture. Conversely, within the subgroup of patients with complications, six patients treated with concomitant allograft use and presenting with calcar loss and four-part fractures did not develop screw cut-out. Of these patients, five presented with one or two concurrent risk factors, including non-anatomical reduction in the humeral calcar, varus malalignment, or suboptimal positioning of the calcar screws.

The Deltoid Tuberosity Index (DTI) as a standalone predictor of complications (*p* = 0.119) was not confirmed by regression analysis. No significant effect of DTI was confirmed for the incidence of AVN (*p* = 0.383), head collapse (*p* = 0.749), or the need for reoperations (*p* = 0.198). Head Fragment Size (HFZ) was not an independent risk factor for the incidence of complications (*p* = 0.325). No effect was confirmed for the incidence of AVN (*p* = 0.413), humeral head collapse (*p* = 0.191), or the need for reoperations (*p* = 0.770) (Table 3).

## 4. Discussion

Proximal humerus fractures are frequent injuries, particularly in osteoporotic patients, and optimal treatment strategies remain controversial. Although most minimally displaced fractures can be managed conservatively, outcomes depend primarily on fracture pattern, displacement, and stability, and no clear consensus exists regarding treatment indication [10]. Poorer functional outcomes have been associated with advanced age, fracture complexity, varus displacement, dorsal head tilt, tuberosity comminution, and medial calcar involvement, all of which should be considered during treatment planning [11].

Conservative management may be successful even in selected three- and four-part fractures; however, complication rates, including nonunion, malposition, avascular necrosis, and particularly humeral head collapse, remain substantial [12]. Surgical treatment of complex fractures, therefore, especially in elderly patients, continues to be debated. Although locking plate osteosynthesis has improved fixation stability, ORIF in patients over 45 years of age is associated with higher complication rates and inferior functional outcomes [13]. Reverse shoulder arthroplasty has gained popularity in this population due to more predictable outcomes, although revision options remain limited.

Despite its limitations, plate osteosynthesis remains a cornerstone for unstable proximal humerus fractures. The Neer classification significantly influences the indication for bone allograft augmentation, which is used more frequently in four-part fractures; however, its clinical benefit in patients younger than 65 years appears limited [14]. Complication and reintervention rates increase with age [15], and reported failures include avascular necrosis, screw cut- out, subacromial impingement, fixation failure, and stiffness, with risk factors related to patient characteristics, fracture morphology, medial cortical integrity, calcar parameters, and surgical technique [16].

Early surgical intervention in our cohort facilitated fracture reduction. Although allograft preparation increases technical demands, failure of osteosynthesis was predominantly associated with patient-related factors, loss of medial support, varus malalignment, fracture complexity, and inadequate reduction and calcar screw placement [17,18]. Allograft use did not significantly affect greater tuberosity reduction, emphasizing the importance of proper plate positioning, stable screw fixation, and secure rotator cuff suturing. While some biomechanical and clinical studies suggest reduced secondary displacement with bone graft augmentation, results remain inconsistent [5,19].

Femoral head allograft augmentation, first described by Euler in 2015, aims primarily to restore medial column support and metaphyseal integrity rather than directly reduce complication rates. In our cohort, decision to use the fresh frozen femoral head allograft was influenced by general indications for its use and also by intraoperative findings. The ultimate decision to use an allograft was made by surgeon, which represents a bias. The indication criteria are presented in the Appendix A Table A1. Disadvantages of using allografts include the risk of transmission of infectious diseases and graft-versus-host reaction, although the incidence of these complications is very low.

In the monitored cohort the femoral head allografts are harvested under sterile conditions during total hip arthroplasty procedures following the patient’s informed consent for tissue donation. Serological testing of blood is performed, and the patient must meet both general and specific eligibility criteria for living tissue or cell donors. Following retrieval, the grafts are decontaminated and transported in a 70% alcohol solution to Central Tissue Bank, then packaged under sterile conditions in laminar airflow cabinet. A swab is obtained from each graft, and a 14-day sterility test is performed in a certified laboratory in accordance with the European Pharmacopoeia. Processed grafts are subsequently frozen at −80 °C and stored under these conditions for up to five years in the Orthopaedic department in the University Hospital Martin.

Although fibular strut allograft is the most commonly used augmentation method, no evidence clearly favors one graft type over another [20]. Fibular grafts provide superior structural support, whereas cancellous femoral head allografts offer better defect filling, remodeling capacity, and do not compromise potential future arthroplasty [2,6,21,22]. Overall complication rates appear comparable. In the literature, we cannot find any RCT studies comparing the results of using fresh frozen femoral head and fibular strut allograft. In the RCT study of 80 patients treated with ORIF using fibular strut allograft augmentation and without its use, its radiological and functional outcomes were comparable [2].

Evaluation of avascular necrosis after plate osteosynthesis is limited by heterogeneous study designs. While plate fixation has been associated with higher AVN rates, this likely reflects fracture complexity and surgical soft tissue disruption rather than fixation method alone [23]. In our cohort, AVN incidence was low and not significantly influenced by allograft use. Hertel’s criteria remain strong predictors of AVN, particularly in fractures involving the calcar region or lesser tuberosity [24], while age alone was not an independent risk factor [5,25].

Importantly, allograft augmentation did not reduce the incidence of humeral head collapse or screw cut-out, which occurred predominantly in four-part fractures with medial calcar disruption. Proposed predictors such as the deltoid tuberosity index remain controversial [26,27]. Higher complication and reoperation rates in the allograft group reflect its preferential use in more complex fracture patterns. Although some studies report favorable outcomes with femoral head allograft augmentation, these benefits are counterbalanced by longer operative time and increased costs [6,28].

Overall, bone allograft augmentation represents a useful adjunct in selected complex proximal humerus fractures, but should not be considered standard treatment. Augmentation techniques may improve construct stability, yet their effectiveness remains dependent on meticulous anatomical reduction, restoration of medial column support, correction of varus alignment, and accurate calcar screw placement [29]. Our findings indicate that allograft augmentation cannot compensate for inadequate surgical technique. Further prospective, randomized studies with long-term follow-up are required to better define patient selection and the true clinical value of graft augmentation.

## 5. Conclusions

In the treatment of proximal humerus fractures, an individual approach to the patient should be preferred, considering risk factors and fracture type according Neer classification.

In patients with proximal humeral fractures treated with PHILOS plate fixation and fresh-frozen femoral head allograft augmentation, the technique was effective, but outcomes were dependent on the reduction and restoration of the medial column support. Mechanical failures occurred predominantly in four-part fractures with initial medial calcar loss. Residual varus malalignment, non-anatomical calcar reduction, and insufficient calcar screw fixation were identified as predictors of secondary loss of stability, despite an allograft use. Consistent with the recent literature, allograft augmentation improves construct stability, but cannot compensate for inadequate reduction or failure to restore medial support, particularly in complex four-part fractures.

In our cohort, none of the risk factors studied showed a significant impact on the development of AVN or the occurrence of pseudoarthrosis. Interruption of the medial hinge, greater tubercle comminution, the use of an allograft augmentation, and medial hinge restoration were identified as significant risk factors for humeral head collapse and screw cut-out. The incidence of secondary dislocation of the greater tubercle was significantly influenced by its comminution, interruption of the medial hinge, and the use of an allograft augmentation. Neither HFZ nor DTI were independent factors influencing the results of surgery and its complications.

A limitation of this study is the uneven distribution of patients with Neer three- and four-part fractures in the groups with and without allograft augmentation. It was also not possible to eliminate the subjective influence of the surgeon when considering the use of an allograft. For this reason, the use of an allograft augmentation in complex proximal humeral fractures requires further expert review.

## Figures and Tables

**Table 1:** Summary of results in relation to the use of a bone graft.

Variables	Overall	PHILOS	PHILOS + FH Allograft	*p*-Value	Statistical Tests
Number of patients	116	84 (72%)	32 (28%)		
Age		69 ± 10.1	65 ± 10.8	*p* = 0.714	χ^2^ test
Sex				*p* = 0.569	Chi-square test
Men	41 (35%)	31	10		
Women	75 (65%)	53	22		
Neer Classification				*p* ˂ 0.001	Chi-square test
3-part	42	39/84 (46%)	3/32 (9%)		
4-part	74	45/84 (54%)	29/32 (91%)		
Dislocation					
Valgus	65 (56%)				
Varus	33 (28%)				
PHILOS− Position					
Optimal	112 (97%)				
Suboptimal	4 (3%)			*p* = 0.906	
Reposition					
Tuberculum majus					
Anatomical	103	75 (89%)	28 (87%)		
Non-anatomical	13	9 (11%)	4 (13%)	*p* = 0.785	χ^2^ test
Calcar humeri					
Anatomical	102	78 (93%)	24 (75%)		χ^2^ test
Non-anatomical	14	6 (7%)	8 (25%)	*p* = 0.008	
Duration of the surgery (min)		86.0 ± 31.9	101.3 ± 21.3	*p* = 0.004	χ^2^ test
Timing of surgery (days)		3.5 ± 2.9	4.7 ± 2.4	*p* = 0.007	WMW-test
Complications					
AVN	4 (3.5%)	2 (2%)	2 (6%)	*p* = 0.307	Chi-square test
Head collapse	9 (8%)	2 (2%)	7 (22%)	*p* = 0.001	Chi-square test
Cut-out	6 (5%)	0 (0%)	6 (19%)	*p* ˂ 0.001	Chi-square test
Screw-loosening	5 (4%)	2 (2%)	3 (9%)	*p* = 0.097	Chi-square test
Reoperation	8 (7%)	4 (5%)	4 (13%)	*p* = 0.142	Chi-square test
Infection	3 (2.5%)	1 (1%)	2 (6%)		
Pseudoathrosis	1 (1%)	1 (1%)	0 (0%)		
Reoperation RSA	6 (5%)	3 (4%)	3 (9%)		

FH allograft—femoral head allograft; AVN—avascular necrosis; RSA—reverse shoulder arthroplasty.

**Table 2:** The influence of risk factors on individual postoperative complications in treatment of proximal humerus fractures.

Observed Variables	Avascular Necrosis			Head Collapse			Cut-Out			Greater Tubercle Redislocation			Nonunion			Infection		
	AVN	NO-AVN	*p*-Value	HC	NO-HC	*p*-Value	Cut-Out	NO-Cut-Out	*p*-Value	GT Redisloc.	NO-GT Redisloc.	*p*-Value	Nonunion	NO-Nonunion	*p*-Value	Infection	NO-Infection	*p*-Value
Age/Years	64 (±5.2)	65.1 (±13.6)	0.867	66.8 (±7.5)	65 (±13.8)	0.697	66.3 (±10.3)	65 (±13.5)	0.818	62.6 (±9.0)	65.2 (±13.5)	0.67	69	65 (±13.4)	0.771	65.7 (±9.1)	65.1 (±13.5)	0.941
Sex			0.091			0.391			0.441			0.824			0.458			0.25
Female	1 (1%)	74 (99%)		7 (9%)	68 (91%)		3 (4%)	72 (96%)		3 (4%)	72 (96%)		1 (1%)	74 (99%)		1 (1%)	74 (99%)	
Male	3 (7%)	38 (93%)		2 (5%)	39 (95%)		3 (7%)	38 (93%)		2 (5%)	39 (95%)		0 (0%)	41 (100%)		2 (5%)	39 (95%)	
Neer Classification of Proximal Humerus Fracture			0.295			0.485			1.000			0.652			1.000			0.552
3 parts fractures	0 (0%)	42 (100%)		2 (5%)	40 (95%)		2 (5%)	40 (95%)		1 (2%)	41 (98%)		1 (2%)	41 (98%)		1 (2%)	41 (98%)	
4 part fractures	4 (5%)	70 (95%)		7 (10%)	67 (90%)		4 (5%)	70 (95%)		4 (5%)	70 (95%)		0 (0%)	74 (100%)		2 (3%)	72 (97%)	
Timing of the Surgery/Days	4.3 (±0.06)	3.8 (±2.9)	0.746	4.4 (±2.4)	3.8 (±2.9)	0.485	5.0 (±2.2)	3.7 (±2.9)	0.291	5.2 (±1.1)	3.8 (±2.9)	0.263	2	3.8 (±2.8)	0.523	6.5 (±0.7)	3.8 (±2.8)	0.176
Interruption of the Medial Hinge/ Calcar Loss			0.944			0.02			0.112			0.027			0.299			0.518
Present	2 (3%)	58 (97%)		8 (13%)	52 (87%)		5 (8%)	55 (92%)		5 (8%)	55 (92%)		0 (0%)	60 (100%)		1 (2%)	59 (98%)	
Missing	2 (4%)	54 (96%)		1 (2%)	55 (98%)		1 (2%)	55 (98%)		0 (0%)	56 (100%)		1 (2%)	55 (98%)		2 (4%)	54 (96%)	
Comminution of Greater Tubercle			0.203			0.005			0.005			0.01			0.257			0.12
Present	3 (6%)	48 (94%)		8 (16%)	43 (84%)		6 (12%)	45 (88%)		5 (10%)	46 (90%)		1 (2%)	50 (98%)		0 (0%)	51 (100%)	
Missing	1 (2%)	64 (98%)		1 (2%)	64 (98%)		0 (0%)	65 (100%)		0 (0%)	65 (100%)		0 (0%)	65 (100%)		3 (5%)	62 (95%)	
Varus-Valgus Deformity			0.799			1.000			0.604			0.356			1.000			1.000
Missing	1 (6%)	17 (94%)		1 (6%)	17 (94%)		0 (0%)	18 (100%)		0 (0%)	18 (100%)		0 (0%)	18 (100%)		0 (0%)	18 (100%)	
Valgus	2 (3%)	63 (97%)		5 (8%)	60 (92%)		5 (8%)	60 (92%)		2 (3%)	63 (97%)		1 (2%)	64 (98%)		2 (3%)	63 (97%)	
Varus	1 (3%)	32 (97%)		3 (9%)	30 (91%)		1 (3%)	32 (97%)		3 (9%)	30 (91%)		0 (0%)	33 (100%)		1 (3%)	32 (97%)	
Bone Graft			0.307			<0.001			<0.001			0.007			0.535			0.125
Allograft augmentation	2 (6%)	30 (94%)		7 (22%)	25 (78%)		6 (19%)	26 (81%)		4 (13%)	28 (87%)		0 (0%)	32 (100%)		2 (6%)	30 (94%)	
Without allograft augmentation	2 (2%)	82 (98%)		2 (2%)	82 (98%)		0 (0%)	84 (100%)		1 (1%)	83 (99%)		1 (1%)	83 (99%)		1 (1%)	83 (99%)	
Medial Hinge Restoration			0.071			0.012			0.002			0.110			1.000			0.038
Calcar anatomical restoration	2 (5%)	100 (98%)		5 (5%)	97 (95%)		2 (2%)	100 (98%)		3 (3%)	99 (97%)		1 (1%)	101 (99%)		1 (1%)	101 (99%)	
No-calcar anatomical restoration	2 (17%)	12 (83%)		4 (29%)	10 (71%)		4 (29%)	10 (71%)		2 (14%)	12 (86%)		0 (0%)	14 (100%)		2 (14%)	12 (86%)	

**Table 3:** DTI and HFZ as independent predictors of complications during plate osteosynthesis for proximal humerus fractures.

Risk Factors	Complications Overall	AVN	Head Collapse	Reoperations
DTI	*p* = 0.119	*p* = 0.383	*p* = 0.749	*p* = 0.198
HFZ	*p* = 0.325	*p* = 0.413	*p* = 0.191	*p* = 0.770

## Data Availability

The original contributions presented in this study are included in the article. Further inquiries can be directed to the corresponding author.

## Abbreviations

The following abbreviations are used in this manuscript:

PHILOS	Proximal Humerus Internal Locking System
RCT	Randomized controlled trial
AVN	Avascular necrosis
DTI	Deltoid Tuberosity Index
HFZ	Head Fragment Size
ORIF	Open Reduction Internal Fixation
RSA	Reverse Shoulder Arthroplasty
AP	Antero-posterior
NaCl	Natriumchlorid
CT	Computer Tomography
GT	Greater Tubercle
FH	Femoral Head

## Appendix A

**Table A1:** Allograft augmentation in proximal humeral fractures [19].

**Indications**	Structural criteria	Poor bone quality (osteoporosis); large epimetaphyseal defect; calcar loss; complex fracture with humeral head impaction.
	Advantages	Mechanical support of the medial column; reduced risk of varus collapse; prevention of secondary displacement.
	Disadvantages	Risk of infection; possible disease transmission; graft-versus-host reaction.
**Contraindications**		Open fractures; oncological diseases treated with chemotherapy; systemic immune disorders; prior graft-versus-host reaction.
